# Nightshift work and risk of breast and prostate cancer: a systematic review and meta-analysis, 2012-2023

**DOI:** 10.23938/ASSN.1149

**Published:** 2025-12-30

**Authors:** Meritxell Soler-Saña, Montserrat Puiggené-Vallverdú, Pere Godoy

**Affiliations:** 1 Hospital Universitari Santa Maria Gestió de Serveis Sanitaris Lleida Spain; 2 Agència de Salut Pública de Catalunya Barcelona Spain; 3 Escola de Doctorat Universitat de Lleida Lleida Spain; 4 Institut de Recerca Biomédica de Lleida [IRBLleida] Universitat de Lleida Lleida Spain; 5 Consorcio de Investigación Biomédica en Red de Epidemiología y Salud Pública [CIBERESP] Madrid Spain

**Keywords:** Breast Neoplasms, Prostatic Neoplasms, Shift Work Schedule, Systematic Review, Occupational Health, Cáncer de Mama, Cáncer de Próstata, Horario de Trabajo por Turnos, Revisión Sistemática, Salud Laboral

## Abstract

**Background:**

The International Agency for Research on Cancer classifies night shift work as Group 2A, a probable human carcinogen. This study updates the evidence on the association between night shift work and the risk of breast and prostate cancer.

**Methods:**

Searches were conducted in PubMed and Web of Science. Case-control and cohort studies were included if they assessed night work as a risk factor for breast or prostate cancer, had ≥100 participants, provided full text in English or Spanish, scored ≥6 on the Newcastle-Ottawa Scale, and reported relative risks or odds ratio with 95% confidence interval. Heterogeneity and study quality were evaluated. Data synthesis followed PRISMA guidelines.

**Results:**

Twenty-one studies including 586,890 participants were analysed. For breast cancer, significant association were found only in cohort studies, both overall (RR=0.82; 95%CI: 0.67-0.99; I^2^=91%) and for <10 years of night work (RR=0.75; 95% CI: 0.68-0.82; I^2^=0%). Average study quality was acceptable (score 7), although heterogeneity was substantial. For prostate cancer, case-control studies reported an OR of 1.14 (95%CI: 1.02-1.27; I^2^=25%). For exposures ≥10 years, both RR (2.20; 95%CI: 1.35-3.59) and OR (1.16; 95%CI: 1.03-1.30) were significant. Overall quality was moderate (score 6) with low heterogeneity (25%).

**Conclusions:**

Weak associations between night shift work and prostate cancer are observed in case-control studies and among individuals with ≥10 years of exposure, but no consistent association was found for breast cancer. These findings remain inconclusive and highlight the need for further research

## RESUMEN

**Fundamento:**

La IARC clasificó el trabajo nocturno como grupo 2A, probable carcinógeno para humanos. Este estudio actualiza la evidencia sobre la relación entre trabajo nocturno y riesgo de cáncer de mama y de próstata.

**Metodología:**

Se realizaron búsquedas en PubMed y Web of Science. Se incluyeron estudios de casos y controles y de cohortes con estos criterios de inclusión: trabajo nocturno como factor de riesgo, muestra >100 personas, texto completo, en inglés o español, puntuación ≥6 en la Escala de Newcastle-Ottawa, y tenían que aportar el riesgo relativo (RR) o la odds ratio (OR) junto con su intervalo de confianza al 95% (IC95%). Se calculó la heterogeneidad (I^2^) y la calidad. La síntesis siguió la guía PRISMA.

**Resultados:**

Los 21 estudios incluidos aportaron 586.890 participantes. Para cáncer de mama, los estudios de cohortes encontraron una relación global débil (RR=0,82; IC95%: 0,67-0,99; I^2^=91%) y, para exposiciones <10 años, una RR=0,75 (IC95%: 0,68-0,82; I^2^=0%), con calidad 7 y alta heterogeneidad. Respecto al cáncer de próstata, los estudios de casos y controles presentaron una OR=1,14 (IC95%: 1,02-1,27) y, para exposiciones ≥10 años, tanto el RR (2,20; IC95%: 1,35-3,59) como la OR (1,16; IC95%: 1,03-1,30) fueron significativos. La calidad global fue 6 y la heterogeneidad fue baja (25%).

**Conclusiones:**

Se observó una relación débil entre trabajo nocturno y cáncer de próstata, en estudios de casos y controles y en ambos tipos de estudios para exposiciones ≥10 años, pero no para cáncer de mama. Estos hallazgos no son concluyentes y se requieren más estudios.

## INTRODUCTION

The International Agency for Research on Cancer (IARC) attributed nearly 10 million deaths and 20 million new cases to cancer in 2022, with lung cancer being the most common in men and breast cancer in women^1^. The causes of cancer are diverse and difficult to define. Risk factors that may increase the likelihood of developing certain types of cancer range from non-modifiable factors, such as age and family history, to exposure to substances like asbestos, or behaviours, including physical inactivity and the consumption of tobacco or alcohol^2^.

In 2007, the IARC classified night shift work (NSW) in Group 2A, as a probable carcinogen for humans^3^^,^^4^. While night shifts may vary in duration and in the number of nights worked per week, month, or year, the International Labour Organisation defined *night work* as work performed over a period of no less than seven consecutive hours, including the interval between midnight and 5 am^5^^-^^8^.

Industries with the highest numbers of night workers include those providing essential services to the population, such as the energy, iron and steel industries, healthcare and caregiving professions, law enforcement, and firefighting^9^. The prevalence of shift work exceeds 15% of the workforce in many countries across North America, continental Europe, and Australia. According to the general report of the sixth European Working Conditions Survey, 19% of workers in the European Union are engaged in night work^10^.

Many biological functions and organic processes such as sleep, digestion, and body temperature follow a cycle that repeats every 24 hours - the circadian rhythm. Melatonin, the hormone that allows our body to synchronise with circadian rhythms, is stored in the pineal gland and begins to be released when the body detects the absence of sunlight. Once released, it initiates a series of consecutive hormonal reactions that conclude in the early hours of the morning as the body begins to restore levels of hormones and other substances depleted during the previous day^11^.

Disruption of circadian rhythms has multiple health effects, including sleep disorders, cardiovascular issues, digestive problems, changes in oestrogen levels associated with certain types of tumours, and psychosocial disturbances^12^^,^^13^. These disruptions may interfere with cellular proliferation, apoptosis, hormonal balance, metabolism, DNA damage and repair, and immune and neuroendocrine functions^14^. In 2020, the IARC dedicated volume 124 of its monographs to NSW, concluding that there is limited evidence regarding the carcinogenicity of night work, although some studies observed associations between night shifts and breast, prostate, colon, and rectal cancers^5^.

However, scientific evidence on the consequences of circadian rhythm disruption and its relationship with cancer remains controversial. Various systematic reviews^9^^,^^11^^,^^15^^-^^20^ report no conclusive association between NSW and breast, prostate, or colon cancer. Given the number of studies with inconsistent results, these reviews have highlighted the need for further research, recommending stronger exposure assessment - as well as more homogeneous exposure variables - and greater control of confounding factors such as menopause or/and chronotypes^20^.

While these literature reviews provided valuable analysis, we consider it appropriate to conduct a more up-to-date review that includes recent studies not covered in earlier publications, and to combine them with previously published studies to achieve sufficient statistical power for a meta-analysis. Our goal is to contribute to the existing body of knowledge by incorporating more recent evidence on this highly relevant topic. This systematic review aims to update the available scientific evidence from 2012 and 2023 on the relationship between NSW and the risk of breast and prostate cancer, using the criteria outlined in the PRISMA 2020 statement, which reflects advancements in methods for identifying, selecting, evaluating, and synthesising studies^21^.

## METHODS

A bibliographic search was conducted between May and June 2024 on the PubMed and Web of Science databases to identify scientific articles published over the past 12 years, from 2012 to 2023. We applied the following comprehensive search strategy: (“Schedule, Shift Work” OR “Schedules, Shift Work” OR “Work Schedule, Shift“ OR “Night Shift Work “ OR “Shift Work, Night” OR “Rota-ting Shift Work” OR “Shift Work, Rotating”) AND (“Tumors” OR “Neoplasia” OR “Neoplasias” OR “Neoplasm” OR “Tumor” OR “Cancer” OR “Cancers” OR “Malignant Neoplasm” OR “Malignancy” OR “Malignancies” OR “Malignant Neoplasms” OR “Neoplasm, Malignant” OR “Neoplasms, Malignant” OR “Benign Neoplasms” OR “Neoplasms, Benign” OR “Neoplasm, Benign” OR “Benign Neoplasm”).

Studies meeting the following inclusion criteria were selected: articles examining night work as a risk factor for breast and prostate cancer, with a sample size ≥100 individuals and availability of the full text. Eligible study designs included cohort studies, case-control studies, and nested case-control studies. Publications in English and Spanish were included. Exclusion criteria comprised studies not meeting the inclusion criteria, duplicate articles across databases, non-original studies, editorials, letters to the editor, books, conference notes, protocols, abstracts and systematic reviews.

Night shifts were defined as night work during the hours between 00:00 and 05:00. Where available, night working life was categorised as ≥10 years (long-term) and <10 years (short-term). In the exposed group, the study population consisted of men and women who worked night shifts, while the control group included men and women who had never worked night shifts. The relationship between cancer diagnosis and long- versus short-term night working life was also evaluated. Studies that did not report night work records, the definition of night work, or the years of exposure were excluded.

Data extraction was performed by two authors who independently reviewed the title and abstract of all search results to identify articles meeting the inclusion criteria and relevant to the research question. Duplicates were removed, and full-text articles potentially eligible for inclusion were retrieved.

A data sheet (table) was designed, in which each of the two researchers independently recorded the following information: study type, eligibility, study characteristics, author, design, study population, evaluation and definition of exposure, number of days, months or years of night work, age at diagnosis, evaluation and definition of outcomes, country where the study was conducted, publication year, measures of association including information on confounding variables (obesity, overweight, alcohol consumption, smoking), exposure group (number of participants), potential stratification, and measures of association - relative risk (RR) or odds ratio (OR) with 95% confidence interval (95%CI) - or the necessary data for their calculation. Reasons for excluding non-included studies were documented.

Each author independently extracted data using the eligibility criteria, the same data sheet, and the same variables. Any disagreements were resolved through discussion or, if needed, by involving a third author.

A pilot assessment was conducted with the first ten included studies to evaluate the suitability of the information collected and the proposed variables. The final data sheet was refined based on suggestions derived from this pilot study.

The methodological quality of the included articles was evaluated using the Newcastle-Ottawa Scale (NOS). The NOS assesses the quality of non-randomised observational studies and incorporates quality and risk-of-bias considerations into the interpretation of meta-analytic results. The scale evaluates three domains: selection and representativeness of study groups, assessment of exposure or outcome, and comparability of groups based on control of potential confounders. A high-quality study should score 3-4 stars in the selection and representativeness domain, 2-3 stars in the exposure/outcome domain, and 1-2 in the comparability dimension^22^^,^^23^.

Each domain was scored with the number of stars deemed appropriate by the research team based on predefined criteria. Higher NOS scores indicated better-quality studies. Studies scoring ≥6 stars were in the data synthesis. Quality assessment was performed by both researchers, resolving disagreements through discussion.

The data synthesis strategy for each phase of the study followed the Preferred Reporting Items for Systematic Review and Meta-Analysis (PRISMA) guidelines and was conducted using Review Manager Web (RevMan Web; The Cochrane Collaboration, 2024). Descriptive tables and graphs were used to present individual study results. Interpretation of the results was based on OR/RR, combined wherever possible, along with the corresponding 95% CIs.

For the statistical analysis, a random-effects model was selected as the I^2^ statistic for heterogeneity was ≥50%. To explore sources of potential heterogeneity, subgroup analyses were performed, stratifying by study type, geographic area, study quality, long-term and short-term exposure, and cancer type. Statistical significance was set at p ≤0.05.

The systematic review protocol was registered in the PROSPERO international prospective database (2024 CRD42024542400). The registration number was obtained on May 13, 2024. This registry aims to provide a comprehensive list of systematic reviews registered at the outset to help prevent duplication and reduce the risk of reporting bias^24^^,^^25^.

## RESULTS

**Figure 1:**
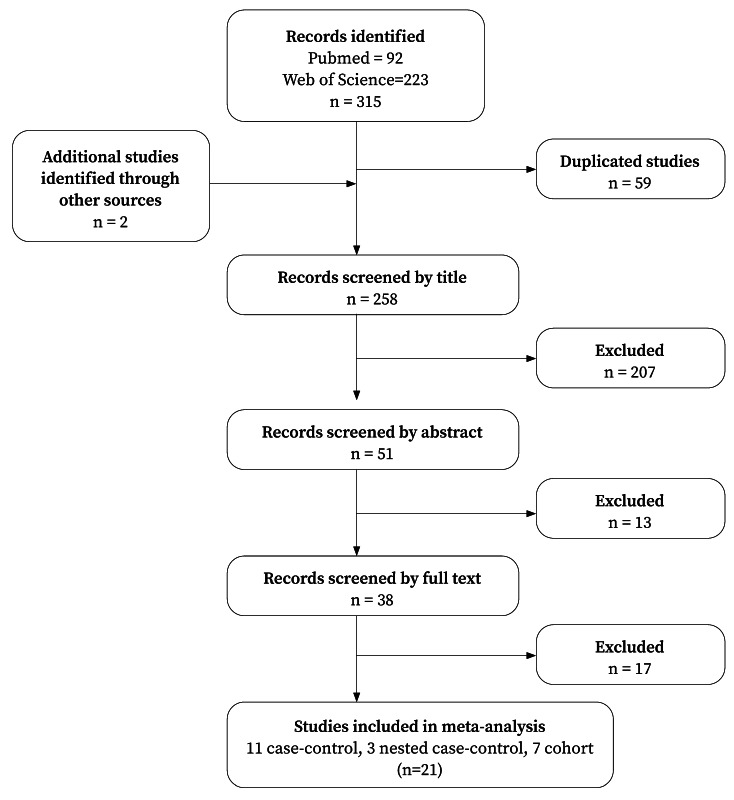
PRISMA flowchart of the study selection process

Through the literature search across the two databases, 315 articles were initially identified. During the bibliographic review, two additional articles were identified and included in the meta-analysis. After removing duplicates, the titles of 258 articles were reviewed, and 207 articles were excluded. The abstracts of the remaining 51 articles were screened, resulting in the exclusion of 13 additional articles. Thus, 38 full-text articles were assessed, of which, 17 were excluded for not meeting the inclusion criteria, leaving 21 final articles. Ultimately, the study consisted of 21 articles (Fig. 1). All the considered studies were published in English.

The 21 included studies were stratified into four subgroups: cohort studies on breast cancer, case-control studies on breast cancer, cohort studies on prostate cancer, and case-control studies on prostate cancer.

We identified a total of 586,890 participants. Geographically, 13 of the 21 studies were conducted in European countries^26^^-^^38^, four from the Americas^39^^-^^42^, three in Asia^43^^-^^45^, and one in Oceania^46^.

### Breast cancer

Fourteen studies examined the relationship between night work and breast cancer: nine case-control studies^33^^,^^34^^,^^38^^,^^39^^,^^42^^-^^46^ and five cohort studies^29^^,^^31^^,^^35^^,^^37^^,^^41^ (Table 1).

**Table 1:** Characteristics of breast cancer and night-shift work studies

Author	Title	N	NSW	Adjustment factors	Quality score
Year		Case	Definition		
Country		Control	Category*		
Case-control studies					
Bustamante-Montes	Night Shift Work and Risk of Breast Cancer in Women	101	9pm-7am	sociodemographic, reproductive, NWS, family history (cancer, BMI), lactation history, smoking, age, type of health insurance, residential status	7
-2019		101			
Mexico			Short/long		
Pham	Night-shift work, circadian and melatonin pathway related genes and their interaction on breast cancer risk: evidence from a case-control study in Korean women	959	regularly	age, pregnancies, education, BMI, smoking, alcohol, family history, menopausal status	6
(2019)		941	9pm-8am		
Korea			Short/long		
Li		Shift work and breast cancer among women textile workers in Shanghai, China	1,709			continously	birth year, age at beginning of follow-up, years of employment in STIB	9
(2015)	4,78		0am-5am					
China			Short/long					
Fritschi	The association between different night shiftwork factors and breast cancer: a case-control study		1,202	0am-5am	age group, menopausal status at recruitment, socioeconomic and remoteness score, education, country of birth, BC family history, number of children, breastfeeding, alcohol, physical activity, BMI, and circadian type, rhythm and flexibility	7		
-2013		1,785				
Australia			Short/long			
Grundy	Increased risk of breast cancer associated with long-term shift work in Canada	1,134	11pm-7am	education, ethnicity, health, medical and reproductive history, family history (cancer), lifestyle, smoking and alcohol consumption, physical activity, occupational and residential histories.	8
-2013		1,179			
Canada			Short/long		
Hansen	Nested case-control study of night shift work and breast cancer risk among women in the Danish military	132	≥1 year	length of education, BMI, alcohol, menopausal status, use of MHT and OC, occupational exposure to radar or EMF, occupational PA, satisfactory influence on job, too high workload and work pace, age at menarche and at menopause, number of childbirths, smoking, occasional sun exposure	8
-2012		505	5pm-9am, not including overtime		
Denmark			Short/long		
Liu		Night shift work, chemical coexposures and risk of female breast cancer in the Norwegian Offshore Petroleum Workers (NOPW) cohort: a prospectively recruited case-cohort study	79			≥7 h including	age, height, weight, BMI, reproductive history, postmenopausal, education, work history	8
-2022	489		0am-5am					
Norwegian			Short/long					
Wang	Night-shift work, sleep duration, daytime napping, and breast cancer risk		661	awake/working	age, education, BMI, marital status, age at menarche, menopausal status, age at menopause, parity, physical activity, breast feeding, BC family history	8		
(2015)		714	0am-6am			
China			*			
Papantoniou		Breast cancer risk and night shift work in a case-control study in a Spanish population	1,708	partly/entirely			age, educational level, family socioeconomic level, race, BMI, BC family history, age of menarche, parity, age at the first birth, menopausal status, smoking, OC, MHT, leisure time physical activity (type, frequency and duration), current sleep duration, sleep problems for ≥1 year (waking up at night, difficult to falling asleep, use of sleep medication), diet habits.	8
(2016)	1,778		0am-6am					
Spain			≥3 nights/ month					
			Short/long					
Cohort studies								
Wegrzyn	Rotating Night-Shift Work and the Risk of Breast Cancer in the Nurses’ Health Studies	78,516	≥3 nights/ month + days/evenings	age, height, current BMI and BMI at 18 years, childhood and adolescent body size, age at menarche and first birth, parity, breastfeeding, age of menopause, MHT, duration of estrogen-only MHT, duration of combined estrogen and progesterone MHT, first-degree family history of BC, history of benign breast disease, alcohol, PA level, mammography use, current smokers, education level, husband education	7			
(2017)		5,971			
USA			*		
Vistisen	Short-term effects of night shift work on breast cancer risk: a cohort study of payroll data	122,755	≥3 h including	age, age first children’s birth, number of children, family history breast cancer, OC, hormones, medications for alcoholism, highest family education, mammography	8
-2016		1,176	0am-5am		
Denmark			Short		
Åkerstedt		Night work and breast cancer in women: a Swedish cohort study	13,656			10pm-6am	Educational level, smoking, alcohol, physical activity, BMI, have children, coffee, previous cancer, menopause, use of hormones, including OC at the time of interview	7
-2015	463		≥1 night/month					
Sweden			Short/long					
Gustavsson	Night work and breast cancer risk in a cohort of female healthcare employees in Stockholm, Sweden		25,585	≥3 h including	age, country of birth, profession, childbirth	6		
-2023		299	11pm-6am			
Sweden			Short/long			
Koppes		Night work and breast cancer risk in a general population prospective cohort study in the Netherlands	285,723	0am- 6am			age, origin, children in the household, education, occupational group, contractual working hours, job tenure	9
-2014	2,531							
Netherland			*					

NSW: night shift work; *: the stratified information could not be provided, and no classification has been made into any category; BMI: body mass index; STIB: Shanghai Textil Industry Bureau; EMF: electromagnetic fields; MHT: menopausal hormonal therapy; OC: oral contraceptive; PA: physical activity.

**Figure 2:**
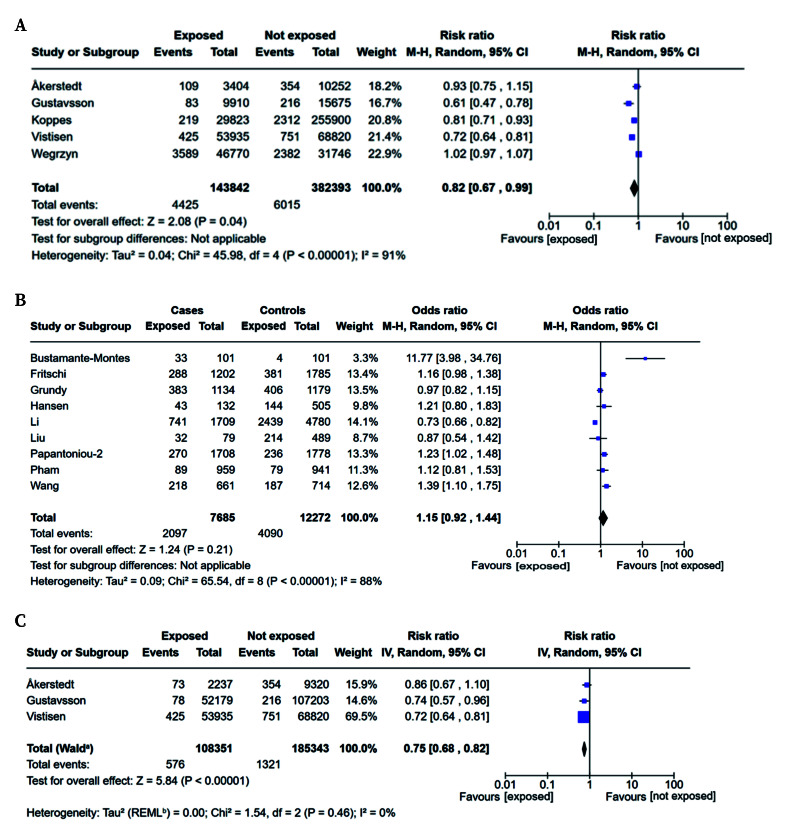
Forest plot for cohort (**A**) and case-control studies (**B**) of overall exposure to night work on the risk of breast cancer. **C.** Forest plot for cohort studies of short-term exposure to night work on the risk of breast cancer.

Cohort studies on breast cancer^29^^,^^31^^,^^35^^,^^37^^,^^41^ included a total of 526,235 participants, of whom 10,440 were breast cancer cases (4,425 in the exposed group and 6,015 in the non-exposed group). These studies reported a pooled RR value of 0.82 (95% CI: 0.67-0.99) (Fig. 2A). Although the reported summary RR showed weak statistical significance, heterogeneity was high (I^2^ = 91%).

Case-control studies on breast cancer included 19,957 participants^33^^,^^34^^,^^38^^,^^39^^,^^42^^-^^46^, of whom 7,685 were cases and 12,272 were controls. The pooled OR was 1.15 (95%CI: 0.92-1.44; I^2^ = 88%) (Fig. 2B).

The exposure to NSW was categorised as short-term (<10 years) in eight case-control studies^33^^,^^34^^,^^38^^,^^39^^,^^42^^-^^44^^,^^46^ and three cohort studies^29^^,^^31^^,^^35^*.* There was no statistically significant association between NSW and breast cancer in case-control studies (OR=0.99; 95%CI: 0.77-1.28) (Supplementary material, figure 1) but cohort studies detected a statistically significant RR=0.75 (95%CI: 0.68-0.82) (Fig. 2C).

Exposure to NSW was categorised as long-term (≥10 years) in eight case-control studies^33^^,^^34^^,^^38^^,^^39^^,^^42^^-^^46^ and two cohort studies^31^^,^^35^. Again, not statistically significant relationship was observed between long-term NSW and breast cancer (OR=1.24; 95% CI: 0.94-1.63 for case-control; RR=1.66; 95% CI: 0.61-4.53 for cohort studies), although both types of studies showed higher risk estimates (Supplementary material, figures 2 and 3).

### Prostate cancer

The remaining seven articles focused on prostate cancer: five case-control studies^26^^,^^27^^,^^32^^,^^36^^,^^40^^)^ and two cohort studies^28^^,^^30^ (Table 2).

**Table 2:** Characteristics of prostate cancer and night-shift work studies

Author	Title	N	NSW	Adjustment factors	Quality score
Year		Case	Definition		
Country		Control	Category*		
*Case-control studies*					
Lozano-Lorca	Night shift work, chronotype, sleep duration, and prostate cancer risk: CAPLIFE study	465	10pm-6am	education, BMI, smoking, physical activity, first-degree family history of prostate cancer, aggressiveness, chronotype, sleep duration	7
(2020)		410			
Spain			Short/long		
Barul	Night-shift work and risk of prostate cancer: results from a Canadian case-control study, the prostate cancer and environment study	1,892	≥3 h including	ancestry, educational level, first-degree family history of prostate cancer	7
(2019)		1,951	0am-5am		
Canada			≥1 year		
			Short/long		
Wendeu-Foyet		Night work and prostate cancer risk: results from the EPICAP Study	818			270 h/year	Gleason score, age, race, family history of prostate cancer, educational level, BMI, physical activity, smoking, alcohol drinking, chronotype, sleep duration.	8
(2018)	875		3 nights/month					
France			≥1 year					
			Short/long					
Papantoniou	Night shift work, chronotype and prostate cancer risk in the MCC-Spain case-control study		1,095	0am-6am	age, participating centre, family history, educational level, BMI, tabaco smoking, past sun exposure, sleep problems, chronotype	8		
(2015)		1,388	3 times/month			
Spain			Short/long			
Berge		Night shift work and risk of aggressive prostate cancer in the Norwegian Offshore Petroleum Workers (NOPW) cohort	2,219	7pm-7am			age, education, anthropometric, BMI, weight, main occupational activity in last position	6
(2023)	280		14 nights/month					
Norwegian			*					
*Cohort studies*								
Behrens	Shift work and the incidence of prostate cancer: a 10-year follow-up of a German population-based cohort study	1,757	≥7 h including	BMI, physical activity, alcohol consumption, education, vitamin D, age	7
(2017)		76	0am-5am		
Germany			Short/long		
Hammer (2015) Germany		Shift work and prostate cancer incidence in industrial workers: a historical cohort study in a German chemical company	27,828			6pm-6am (12h)	professional status, type activity, smoking, duration employment, living or deceased	6
	337		every 3 or 4 days					
			*					

NSW: night shift work; *: the stratified information could not be provided, and no classification has been made into any category; BMI: body mass index.

**Figure 3:**
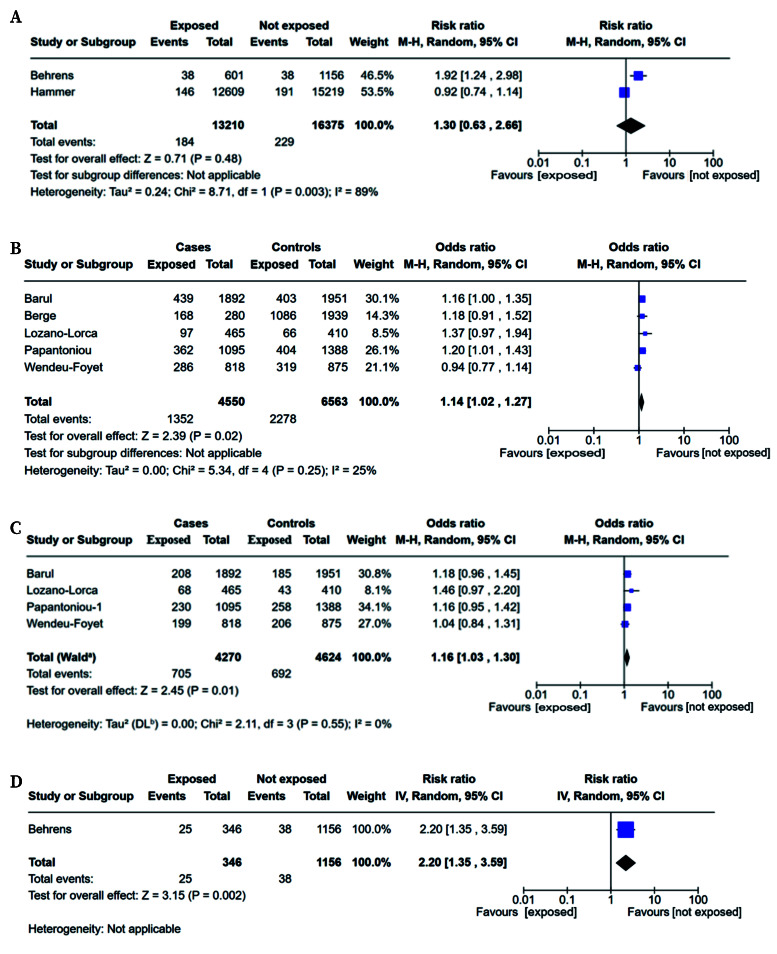
Forest plot for cohort (**A,D**) and case-control studies (**B,C**) assessing the risk of exposure to night work on the risk of prostate cancer. **A**,**B**. Overall. **C**,**D**. Long-term exposure to night work.

Cohort studies on prostate cancer^28^^,^^30^ included 29,585 participants, of whom 413 were cases (184 in the exposed group and 229 in the non-exposed group). These studies reported a non-significant pooled RR value of 1.30 (95%CI: 0.63-2.66; I^2^ = 89%) (Fig. 3A).

Case-control studies on prostate cancer^26^^,^^27^^,^^32^^,^^36^^,^^40^, included 11,113 participants, comprising 4,550 cases and 6,563 controls. The pooled OR was 1.14 (95%CI: 1.02-1.27; I^2^ = 25%) (Fig. 3B). The pooled estimate indicate a statistically significant increase in risk associated with night work, with moderate heterogeneity (I^2^ = 25%).

The exposure to NSW was categorised as short-term (<10 years) in four case-control studies^26^^,^^27^^,^^32^^,^^40^, which showed no statistically significant association (OR=1.04; 95%CI: 0.89-1.21) and in one cohort study^28^, which showed no significance (RR=1.61; 95%CI: 0.87-2.97) (Supplementary material, figures 4 and 5). 

However, when examining the four case-control studies that classified NSW exposure as long-term^26^^,^^27^^,^^32^^,^^40^, a statistically significant association was found (OR=1.16; 95%CI: 1.03-1.30) (Fig. 3C), as well as in the one cohort study^28^ (RR=2.20; 95%CI: 1.35-3.59) (Fig. 3D).

### Quality

The overall quality of the studies ranged from 6 to 9 points on the NOS. The weakest aspect was the exposure/outcome domain. Four studies scored 6 points^30^^,^^35^^,^^36^^,^^43^, seven 7 points^26^^,^^28^^,^^31^^,^^39^^-^^41^^,^^46^, eight 8 points^27^^,^^29^^,^^32^^-^^34^^,^^38^^,^^42^^,^^45^, and two studies scored 9 points^37^^,^^44^. The median quality score for both cohort and case-control studies was 7. For breast cancer studies, the average NOS score was 7, representing overall acceptable quality. For prostate cancer, the average score was 7 for case-control studies and 6 for cohort studies, suggesting slightly higher quality among the case-control studies.

### Heterogeneity

Heterogeneity was high, indicating limitations. Table 3 presents pooled risk estimates for each cancer type and corresponding heterogeneity values.

For the breast cancer studies, heterogeneity in the short term subgroup was high for case-control studies and 0 for cohort studies. For the long-term subgroup, heterogeneity exceeded 50% for both study designs. For prostate cancer case-control studies, heterogeneity was moderate for short-term analyses and 0 for long-term analyses; for cohort studies, heterogeneity could not be assessed.

**Table 3:** Pooled risk estimates for breast and prostate cancer and heterogeneity analysis

	Study design	Studies n (references)	OR/RR (95%CI)	I^2^ (%)	p-value
*Breast cancer*	*Short-term (<10years) versus never night-shift work*				
	Case control	8^32^^,^^33^^,^^37^^,^^38^^,^^41^^,^^42^^,^^43^^,^^45^	0.99	67	0.96
			(0.77-1.28)		
	Cohort	3^28^^,^^30^^,^^34^	0.75	0	<0.001
			(0.68-0.82)		
	*Long-term (>10 years) versus never night-shift work*				
	Case control	8^32^^,^^33^^,^^37^^,^^38^^,^^41^^,^^42^^,^^43^^,^^45^	1.24	85	0.13
			(0.94-1.63)		
	Cohort	2^30^^,^^34^	1.66	78	0.32
			(0.61-4.53)		
*Prostate cancer*	*Short-term (<10years) versus never night-shift work*				
	Case control	4^25^^,^^26^^,^^31^^,^^39^	1.04	21	0.65
			(0.89-1.21)		
	Cohort	1^27^	1.61	-	0.13
			(0.87-2.97)		
	*Long-term (>10 years) versus never night-shift work*				
	Case control	4^25^^,^^26^^,^^31^^,^^39^	1.16	0	0.01
			(1.03-1.30)		
	Cohort	1^27^	2.20	-	0.002
			(1.35-3.59)		

OR: *odds ratio*; RR: relative risk; CI: confidence interval; I^2^: heterogeneity measure.

## DISCUSSION

This meta-analysis included 21 studies with 20,670 cancer cases and 586,890 participants, and found no association between NSW and either breast or prostate cancer in the specific analyses.

When individually analysing the studies included in our meta-analysis, only three show a statistically significant relationship between NSW and breast cancer^38^^,^^39^^,^^45^, and three do so for prostate cancer^28^^,^^32^^,^^40^, although all report weakly associations. Eleven studies found no conclusive results for breast cancer^29^^,^^31^^,^^33^^-^^35^^,^^37^^,^^41^^-^^44^^,^^46^ and four reported inconclusive findings for prostate cancer^26^^,^^27^^,^^30^^,^^36^.

Among the three studies that reported an association between breast cancer and NSW^38^^,^^39^^,^^45^, it is noteworthy that they supported the hypothesis that certain factors involved in night work may be linked to increased breast cancer risk. However, due to several limitations (e.g., sample size, insufficient information on work schedules or confounding factors such as alcohol use or ethnicity), the observed associations are weak and remain controversial. All three were case-control studies; none of the cohort studies found an association. It is important to note that case-control studies may provide more precise estimates when the outcome under study has low prevalence, as is common in cancer research^20^^,^^59^. The authors recommend further population-based studies to confirm these findings, particularly in diverse professionals and ethnic groups.

Regarding studies with inconclusive results, Li *et al.*^44^ limited to textile workers in Shanghai and suggested possible differences between Caucasian and Asian women, Liu *et al*.^34^ evaluated a small cohort of offshore oil workers, and Vistisen *et al*.^29^ focused on short-term exposure. This last study found a significant protector effect of short-term exposition to NSW on breast cancer. Due to the weight of its sample size on our results, the protector effect detected in this meta-analysis should be interpreted with caution.

Jones *et al*.^60^, excluded from our meta-analysis due to analytical incompatibility, also found no association between NSW and breast cancer in the Generations Study cohort. Among 102,869 women recruited between 2003 and 2014, followed for a median of 9.5 years, 2,059 developed invasive breast cancer. The hazard ratio for NSW was 1.00 (95%CI: 0.86-1.15).

Regarding prostate cancer, our meta-analysis found that Barul *et al.*^40^ suggested weak positive associations for forward-rotating night shifts with high rotation frequency. Behrens *et al.*^28^ identified an increased risk for long-term exposure and among men with an early preferred midpoint of sleep. Papantoniou *et al.*^32^ reported differential effects by chronotype and suggested a possible role of night work in prostate cancer risk. Likewise, Lozano-Lorca *et al.*^26^ observed a higher risk among workers with an evening chronotype. Moreover, both case-control and cohort studies in our meta-analysis indicate a statistically significant risk of prostate cancer for exposures longer than 10 years. In line with this, the meta-analysis by Rao *et al.*^61^ (2015) reported a 2.8% increase in prostate cancer risk for every 5 years of night work exposure.

It is important to note that in several studies, prostate cancer cases were classified as incident when they were actually prevalent, complicating the assessment of a causal relationship between NSW and prostate cancer.

Multiple meta-analyses have investigated the relationship between NSW and breast cancer. The first, published in 2005 by Megdal *et al*.^47^ found an association (RR=1.51; 95% CI: 1.36-1.68), although it included few studies. In 2008, Erren *et al*.^48^ also linked chronodisruption to cancer, particularly in flight personnel. However, studies published in 2013 showed contradictory results: Ijaz *et al.*^49^, Jia *et al.*^50^, and Kamdar *et al*.^51^ reported high heterogeneity between studies, and Wang *et al.*^52^ suggested a positive association but emphasized inconsistency in exposure definitions. Later studies, including He *et al*.^53^ and Lin *et al.*^54^ (2015), concluded that NSW increases breast cancer risk, whereas Travis *et al.*^55^ reported little or no effect. Liu *et al.*^15^ (2018) identified a positive association with no sex difference and noted an increased risk with cumulative years of night work. Manouchehri *et al.*^9^ observed positive results for short-term but not long-term night shift workers, with association in subgroups such as flight attendants on long-haul overnight routes. They recommend further research for more robust conclusions. In the same year, Van *et al*.^56^ analysed 32 studies and concluded there was no association. A recent update meta-analysis by Wei *et al*.^17^ found a significant increased risk of breast cancer among women with more than ten years of night-shift work. Hansen *et al.*^20^ concluded that newer findings were consistent with the 2019 IARC evaluation and do not provide additional evidence, they recommended studies with better exposure assessment and inclusion of relevant interactions, such as menopausal status, chronotype, tumour hormonal subtype, and genetic-environmental interactions.

Meta-analyses on prostate cancer and NSW are fewer, also showing inconsistent results. The first, by Erren *et al.*^48^, reported an association. Du *et al.*^57^ (2017) found no clear association analysing cohort studies, although subgroup analyses suggested increase risk among Asian men. Mancio *et al.*^58^ (2018) reported a slightly higher risk among rotating night shift workers, although weak. Gan *et al.*^16^ found that shift work was significantly associated with prostate cancer and identified a non-linear relationship between duration and risk. Rivera-Izquierdo *et al.*^18^ concluded that current evidence remains insufficient to confirm an association. Additional analytical studies using more objective and homogeneous exposure measures are necessary.

Overall, these meta-analyses show conflicting results for breast cancer and NSW, and further studies incorporating menopausal status and hormonal markers are needed. However, case-control and cohort studies with long-term exposure suggest a possible association between NSW and prostate cancer.

One strength of our study is the updated literature search, incorporating recent publications not included in earlier systematic reviews and meta-analyses. Specifically, one study^36^^)^ included in the breast cancer analysis and one^26^ in the prostate cancer analyses were not incorporated in previous meta-analyses.

Our study has several limitations. First, the definition of NSW varies across studies and countries. Most studies defined NSW as ≥ 3 hours of work between midnight and 5am.^26^^,^^28^^,^^29^^,^^31^^,^^32^^,^^34^^-^^37^^,^^40^^,^^42^^,^^44^^-^^46^. Second, many studies (43%) assessed exposure using self-reports or non-blinded interviews in case-control studies^32^^,^^38^^-^^40^^,^^42^^,^^43^^,^^46^ and self-reports or poorly described methods in cohort studies^28^^,^^41^, potentially leading to recall and misclassification bias. Third, the classification of short- and long- term could not be uniformly applied, as some studies did not stratify by exposure duration. Fourth, high heterogeneity was observed in the meta-analysis of all studies. Fifth, breast cancer is among the most biologically heterogeneous cancers, and some authors argue that triple-negative tumours are not comparable to hormone-receptor-positive cancers; however, most studies do not provide stratified results. Sixth, obesity, alcohol consumption, and smoking may be in the casual pathway if night work negatively affects lifestyle. Adjusting for these factors could have artificially reduced observed associations. Seventh, although the IARC classification of NSW as a probable carcinogen provides a causal framework, methodological limitation in the underlying studies prevent proper evaluation of causal relationships with breast or prostate cancer.

This meta-analysis does not identify an association between breast cancer and NSW, whereas both case-control and cohort studies suggest a statistically significant increased risk of prostate cancer for exposures longer than ten years. Nonetheless, discrepancies persist. Differences may result from non-comparable populations in terms of age, sociodemographic characteristics (e.g., geographic region, healthcare systems), or occupational characteristics (e.g., cohort limited to specific sectors). The lack of a uniform definition of NSW, variation in exposure assessment, inconsistent treatment of confounders (e.g., some studies not adjusting for age), and differences in follow-up also hinder accurate risk estimation.

These findings are consistent with recent studies reporting inconclusive evidence regarding the relationship between night and shift work and breast and prostate cancer. Despite our results, a potential association cannot be ruled out, and we conclude that further studies are needed.

## Data Availability

They are available upon request to the corresponding author.
